# *Staphylococcus aureus* Leukocidin LukED and HIV-1 gp120 Target Different Sequence Determinants on CCR5

**DOI:** 10.1128/mBio.02024-16

**Published:** 2016-12-13

**Authors:** Kayan Tam, Megan Schultz, Tamara Reyes-Robles, Bénédicte Vanwalscappel, Joshua Horton, Francis Alonzo, Beili Wu, Nathaniel R. Landau, Victor J. Torres

**Affiliations:** a Department of Microbiology, New York University School of Medicine, New York, New York, USA; b CAS Key Laboratory of Receptor Research, Shanghai Institute of Materia Medica, Chinese Academy of Sciences, Pudong, Shanghai, China

## Abstract

Leukocidin ED (LukED) is a bicomponent pore-forming toxin produced by *Staphylococcus aureus* that lyses host cells by targeting the chemokine receptors CC chemokine receptor type 5 (CCR5), CXCR1, CXCR2, and DARC. In addition to its role as a receptor for LukED, CCR5 is the major coreceptor for primary isolates of human immunodeficiency virus type 1 (HIV-1) and has been extensively studied. To compare how LukED and HIV-1 target CCR5, we analyzed their respective abilities to use CCR5/CCR2b chimeras to mediate cytotoxicity and virus entry. These analyses showed that the second and third extracellular loops (ECL) of CCR5 are necessary and sufficient for LukED to target the receptor and promote cell lysis. In contrast, the second ECL of CCR5 is necessary but not sufficient for HIV-1 infectivity. The analysis of CCR5 point mutations showed that glycine-163 is critical for HIV-1 infectivity, while arginine-274 and aspartic acid-276 are critical for LukED cytotoxicity. Point mutations in ECL2 diminished both HIV-1 infectivity and LukED cytotoxicity. Treatment of cells with LukED did not interfere with CCR5-tropic HIV-1 infectivity, demonstrating that LukED and the viral envelope glycoprotein use nonoverlapping sites on CCR5. Analysis of point mutations in LukE showed that amino acids 64 to 69 in the rim domain are required for CCR5 targeting and cytotoxicity. Taking the results together, this study identified the molecular basis by which LukED targets CCR5, highlighting the divergent molecular interactions evolved by HIV-1 and LukED to interact with CCR5.

## IMPORTANCE

The bicomponent pore-forming toxins are thought to play a vital role in the success of *Staphylococcus aureus* as a mammalian pathogen. One of the leukocidins, LukED, is necessary and sufficient for lethality in mice. At the molecular level, LukED causes cell lysis through binding to specific cellular receptors. CCR5 is one of the receptors targeted by LukED and is the major coreceptor for CCR5-tropic HIV-1. While the molecular interaction of CCR5 and HIV-1 is well characterized, the means by which LukED interacts with CCR5 is less clear. In this study, we demonstrated that receptor specificity is conferred through unique interactions between key domains on CCR5 and LukE. Although HIV-1 and LukED target the same receptor, our data demonstrated that they interact with CCR5 differently, highlighting the molecular complexity of host-pathogen interactions.

## INTRODUCTION

*Staphylococcus aureus* and human immunodeficiency virus (HIV) are formidable human pathogens. Multidrug-resistant *S. aureus* is estimated to cause over 80,000 cases of invasive disease annually in the United States, with an increasing prevalence of community-acquired *S. aureus* affecting both immunocompetent and immunocompromised individuals (1, 2). HIV is estimated to affect 1.2 million individuals in the United States (3) and 36.9 million individuals globally (4), causing immunocompetent individuals to become immunocompromised. Studying the commonalities and differences between *S. aureus* and HIV can provide insights into host-pathogen interactions and how these pathogens subvert human hosts.

CC chemokine receptor type 5 (CCR5) is used by both pathogens to mediate virulence. In *S. aureus*, the bicomponent pore-forming toxin leukocidin ED (LukED) mediates immune cell lysis through binding to CCR5 (5). In HIV-1, CCR5 is a coreceptor for infection (6–10). The binding of the viral glycoprotein gp120 to the receptors, CD4 and CCR5, reveals a conserved domain, gp41, thus allowing viral envelope fusion to host cells (11–13). Furthermore, the importance of CCR5 in HIV-1 pathogenesis is highlighted by the naturally occurring Δ32 mutation, a mutation that affects CCR5 surface exposure, rendering individuals with the mutation largely protected from CCR5-tropic HIV-1 infections (14).

In this study, we investigated the molecular basis of the interaction between LukED and CCR5 using CCR5/CCR2b chimeric receptors, an approach that has been exploited previously for studying gp120-CCR5 interactions (15, 16). We identified extracellular loop 2 (ECL2) and ECL3 of CCR5 to be necessary and sufficient for LukED-mediated cytotoxicity. In contrast, for HIV-1, ECL2 of CCR5 is necessary but not sufficient to mediate fusion by gp120. We also defined the domains within LukE that target CCR5. Our study revealed that while gp120 and LukED both target CCR5, they bind to different regions of the receptor, highlighting the divergence of the pathogens. The identification of the structure and function relationship between CCR5 and LukED will add to our understanding of how *S. aureus* leukocidins target cells to form pores, thus improving future assay development and therapeutics against these important toxins.

## RESULTS

### Generation and validation of CCR5 and CCR2b chimeras.

**FIG 1:**
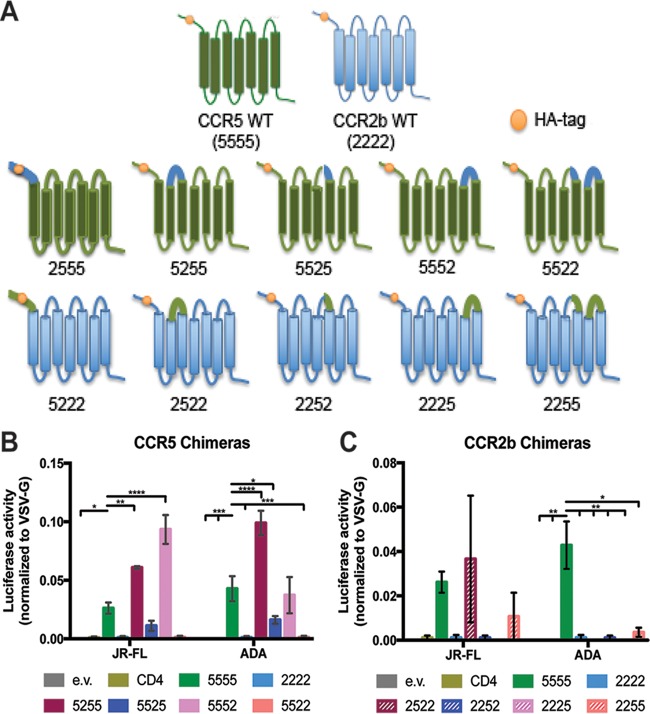
ECL2 of CCR5 is necessary but not sufficient for CCR5-tropic HIV-1 infection. (A) Models representing the CCR5/CCR2b chimeras used in this study. Blue represents CCR2b, and green represents CCR5. All engineered wild-type (WT) and chimeric receptors contained a HA tag at the N terminus (orange circle). Chimeric receptors are named based on the parentage of their extracellular domains in the following order: N terminus, extracellular loop 1, extracellular loop 2, extracellular loop 3. “5” indicates the extracellular domain belonging to CCR5; “2” indicates the extracellular domain belonging to CCR2b. (B and C) HEK293T were cells transfected to express CD4 and the CCR5 chimeric receptors (B), the CCR2 chimeric receptors (C), or empty vector (e.v.) following incubation with JR-FL-, ADA-, and VSV-G-pseudotyped reporter virus. Bars represent the mean levels of luciferase activity normalized to the VSV-G control ± standard errors of the means (SEM) (*n* = 3). Statistical analyses were performed using two-way ANOVA with Dunnett’s multiple comparison. *, *P* < 0.05, **, *P* < 0.01, ***, *P* < 0.001, ****, *P* < 0.0001.

CCR5 and CCR2b share 79% amino acid identity (15). To understand the molecular basis of the interaction between LukED and CCR5, we adapted the CCR5/CCR2b chimeric receptor system used to identify the interactions between gp120 and CCR5. Previous CCR5/CCR2b chimeras swapped entire segments of CCR5/CCR2b (15, 16), including the transmembrane and intracellular domains. Some of these domains were found to be critical for ligand binding and receptor signaling (17, 18). To discriminate more finely, we designed chimeric receptors in which only the extracellular domains were swapped (Fig. 1A), thereby minimally perturbing the sequence of the receptor for studying the contribution of LukED targeting without disrupting the transmembrane and intracellular domains.

To confirm the functionality of the CCR5/CCR2b chimeras, we coexpressed CD4 and the chimeric receptors in transiently transfected HEK293T cells and then infected them with single-cycle HIV-1 luciferase reporter viruses pseudotyped with two different CCR5-tropic HIV-1 envelope glycoproteins, namely, JR-FL and ADA, and the vesicular stomatitis virus G protein (VSV-G) as a positive control that infected all the cells (Fig. 1B and C). After 3 days, we measured intracellular luciferase activity. HEK293T cells coexpressing CD4 and CCR5 were infected with HIV, while those transfected with empty vector, CD4 alone, or CD4 and CCR2b remained uninfected (Fig. 1B and C).

We next evaluated how the chimeric receptors affect HIV-1 infectivity. When ECL1 or ECL3 of CCR5 was swapped with the corresponding ECLs of CCR2b (CCR5 chimeric receptors 5255 and 5552, where “5” indicates the location of CCR5 sequences and “2” indicates the location of CCR2b sequences), there was no defect in HIV-1 infectivity (Fig. 1B). Swapping out ECL2 of CCR5 (5525) resulted in an approximately 60% decrease in the levels of JR-FL-infected cells and ADA-infected cells compared to the CCR5 wild type (WT) (5555) (Fig. 1B). Swapping out both ECL2 and ECL3 resulted in complete protection from both JR-FL and ADA (Fig. 1B). In contrast, ECL2 from CCR5 in CCR2b (CCR2b chimeric receptors 2252 and 2255) was not sufficient to render the CCR2b chimera-expressing cells susceptible to HIV-1 infection (Fig. 1C). Taken together, these data demonstrated that the chimeric receptors behave similarly to previously reported chimeric receptors generated by domain swapping (15).

### CCR5 ECL2 and ECL3 are necessary and sufficient to render cells susceptible to LukED.

**FIG 2:**
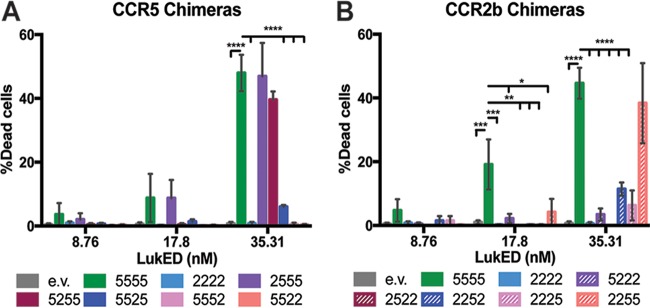
ECL2 and ECL3 of CCR5 are necessary and sufficient for LukED cytotoxicity. Viability of 293T cells expressing the CCR5 chimeric receptors (B) and the CCR2 chimeric receptors (C) following incubation with increasing concentrations of LukED was analyzed. Cell viability was determined by membrane permeability using eFluor450 fixable viability dye. *n* = 3; data represent mean percentages of dead cells ± SEM. Statistical analyses were performed using two-way ANOVA with Dunnett’s multiple comparison. *, *P* < 0.05, **, *P* < 0.01, ***, *P* < 0.001, ****, *P* < 0.0001.

We next elucidated the receptor sequence determinants required for the interaction between LukED and CCR5. We hypothesize that if LukED specificity for CCR5 were mediated by the extracellular domains, then LukED would lose its ability to associate with the chimeric receptors without the relevant extracellular regions. To test the hypothesis, we transfected HEK293T cells with expression vectors for WT or chimeric receptors and then subjected them to LukED intoxication. All the chimeric receptors were expressed and detected on the surface of the cells (see Fig. S1 in the supplemental material). Following LukED intoxication, cells expressing CCR5 chimeric receptors containing the N terminus or ECL1 substitutions (2555 and 5255) showed a level of cytotoxicity similar to that seen with cells expressing WT CCR5 (5555) (Fig. 2A). In contrast, the CCR5 chimera containing an ECL2 substitution (5525) resulted in protection compared to 5555. Similarly, chimeric receptors containing substitution of ECL3 (5552) or substitution of ECL2 and ECL3 (5522) resulted in complete protection (Fig. 2A).

Next, we evaluated if ECL2 and ECL3 of CCR5 are sufficient to confer susceptibility to CCR2b-expressing HEK293T cells. Substitution of the N terminus and ECL1 of CCR5 to CCR2b (5222, 2522) produced results similar to those seen with WT CCR2b (2222). While ECL2 or ECL3 substitution of CCR5 to CCR2b (2252, 2225) resulted in a modest increase in susceptibility, the replacement of both ECL2 and ECL3 (2255) phenocopied WT CCR5 (5555) intoxication by LukED (Fig. 2B). Taken together, our data demonstrated that ECL2 and ECL3 of CCR5 are necessary to render CCR5-expressing cells susceptible to LukED and are sufficient to render an otherwise incompatible chemokine receptor, CCR2b, susceptible to LukED.

### CCR5 ECL2 and ECL3 are involved in LukED interaction with host cells.

**FIG 3:**
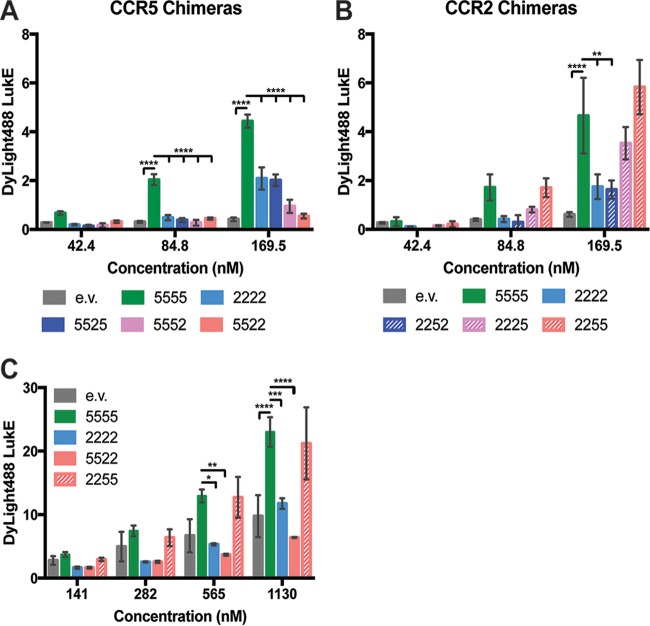
LukE associates with the cell membrane through binding to ECL2 and ECL3 of CCR5. (A and B) Levels of LukE binding to cell surfaces of HEK293T cells transfected with CCR5 chimeric receptors (A) and CCR2 chimeric receptors (B) were measured using DyLight488-LukE in complex with WT LukD. (C) Levels of LukE binding to HEK293T cells transfected with 5555, 2222, 5522, and 2255 were measured using DyLight488-LukE in complex with the pore formation-deficient LukD^DN^ mutant. *n* = 3; data represent median fluorescent intensity (MFI) levels ± SEM. Statistical analyses were performed using two-way ANOVA with Dunnett’s multiple comparison. *, *P* < 0.05, **, *P* < 0.01, ***, *P* < 0.001, ****, *P* < 0.0001.

To correlate cell death to toxin binding to the receptors, we measured the levels of LukE association with the surface of CCR5-expressing HEK293T cells. We have previously shown that LukD enhances binding of LukE to host cells (5); thus, we used WT LukD and DyLight488-LukE to detect toxin association with the chimeric receptors. We found that CCR5 chimeric receptors containing substitutions of ECL2 and/or ECL3 (5525, 5552, and 5522) resulted in decreased toxin binding (Fig. 3A). These results correlated to reduced susceptibility to LukED-mediated cell death (Fig. 2A). While we observed LukE binding to CCR2 WT (2222) and the CCR2 chimeric receptor containing ECL2 of CCR5 (2252) at similar levels (Fig. 3B), the CCR2 chimeric receptor containing ECL3 of CCR5 (2225) showed increased LukE binding. LukE was observed to bind the CCR2 chimeric receptor containing both ECL2 and ECL3 of CCR5 (2255) at a level similar to that seen with WT CCR5 (5555) (Fig. 3B), data consistent with the cytotoxicity phenotypes described in Fig. 2B.

An important caveat concerning the use of WT pore-forming toxins in binding assays is that the intoxication dosage must be carefully titrated to minimize cell death while maximizing the binding signal. To circumvent the cytotoxicity aspect of LukED and to try to enhance the DyLight488-LukE signal, we used a LukD pore formation-deficient mutant (LukD^DN^) (19) and DyLight488-LukE to detect LukE association with the chimeric receptors. The noncytotoxic aspect of LukD^DN^ allowed the use of a higher concentration of DyLight488-LukE, which enhanced the binding signal by 5-fold (Fig. 3C). Whole-cell binding assays using LukED^DN^ further demonstrated that both ECL2 and ECL3 of CCR5 are required for maximum binding and cytotoxicity of LukED.

### Transmembrane and ECL2 mutations on CCR5 prevent CCR5-tropic HIV-1 infection.

**FIG 4:**
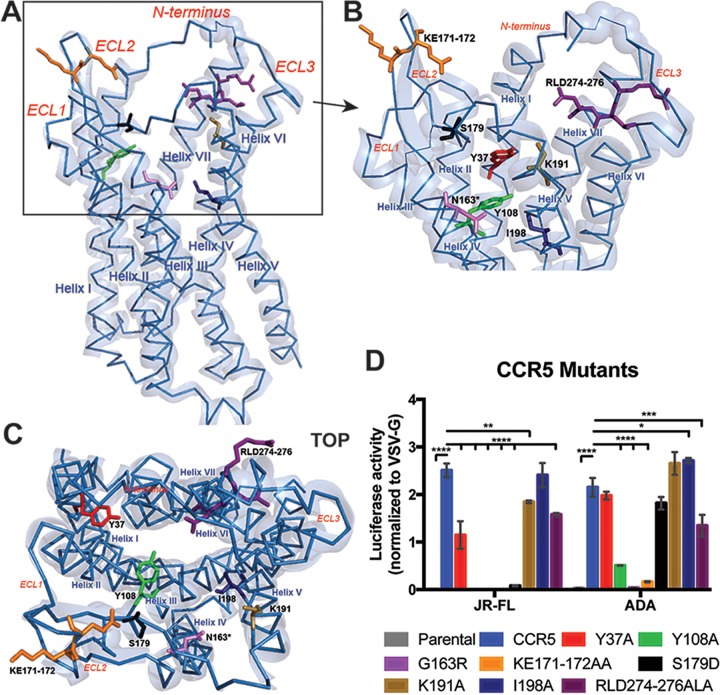
Mutations on CCR5 prevent CCR5-tropic HIV-1 infection. (A) The CCR5 crystal structure (PDB 4MBS) represented as a ribbon in light blue (23). (B) Magnified view of CCR5 showing the locations of the mutations. (C) Top view of the extracellular side of CCR5. Locations of disulfide bonds are represented as faded spheres located between the N terminus and ECL3, ECL1, and ECL2. Mutations used in this study are shown as sticks as follows: Y37 (red), Y108 (green), N163 (pink; glycine at position 163 of the WT CCR5 was mutated to asparagine in this crystal structure [23]), K171-E172 (orange), S179 (black), K191 (beige), I198A (dark blue), and R274-D276 (purple). (D) SupT1 cells expressing CCR5 mutants following incubation with JR-FL-, ADA-, and VSV-G-pseudotyped reporter virus. Bars represent the mean levels of luciferase activity normalized to VSV-G control ± SEM (*n* = 6). Statistical analysis was performed using two-way ANOVA with Dunnett’s multiple comparison. *, *P* < 0.05, **, *P* < 0.01, ***, *P* < 0.001, ****, *P* < 0.0001.

Previous studies defined the amino acid residues on CCR5 that alter HIV-1 susceptibility, antagonist binding, and natural ligand binding (16–18, 20, 21). Based on these findings, CCR5 mutants were tested for coreceptor function using CCR5-tropic JR-FL- and ADA-pseudotyped HIV-1 luciferase reporter viruses. For these experiments, CD4-positive SupT1 cells were transduced with lentiviral vectors that stably express WT CCR5 or CCR5 mutants (Table 1 and Fig. 4A to C; see also Fig. S2). A CCR5 helix IV mutation at G163 and ECL2 mutations at K171 and E172 abolished coreceptor function (Fig. 4D). A helix III mutation at Y108 and an ECL2 mutation at S179 resulted in protection from JR-FL infection; ADA infectivity was severely attenuated with the Y108 mutation, but the S179 mutation had no effect (Fig. 4D). Alanine mutations at Y37 (helix I) and K191 (helix V) resulted in decreased JR-FL infectivity, but ADA infectivity remained similar to that seen with WT CCR5. A helix V mutation at I198 did not affect CCR5-tropic HIV infectivity (Fig. 4D). Alanine mutations at R274 and D276 (helix VII) resulted in a 40% decrease in HIV infectivity in both JR-FL and ADA (Fig. 4D).

**TABLE 1:** SupT1 CCR5 mutants and the locations of the mutations

SupT1 CCR5 mutant	Location of mutation
Y37A	Helix I
Y108A	Helix III
G163R	Helix IV
KE171-172AA	ECL 2
S179D	ECL 2
K191A	Helix V
I198A	Helix V
RLD274-276ALA	Helix VII

### Mutations in ECL2 and helix VII of CCR5 prevent LukED intoxication.

**FIG 5:**
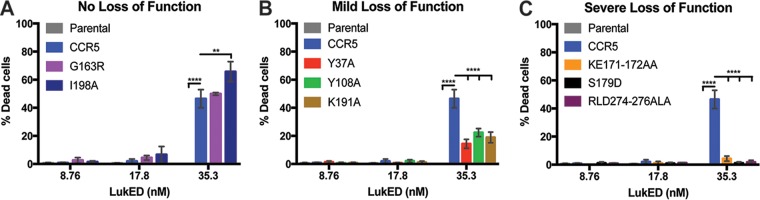
Mutations on ECL2 and helix VII of CCR5 prevent LukED intoxication. The viability of SupT1 cells expressing CCR5 mutants following incubation with increasing concentrations of LukED was determined. Cell viability was determined by membrane permeability using eFluor450 fixable viability dye. *n* = 3; data represent mean percentages of dead cell ± SEM. Mutations with phenotypes of no loss of function (A), mild loss of function (B), and severe loss of function (C) compared to WT CCR5 are indicated. Statistical analyses were performed using two-way ANOVA with Dunnett’s multiple comparison. **, *P* < 0.01, ****, *P* < 0.0001.

Next, we evaluated the effect of the CCR5 mutations on LukED cytotoxicity. The CCR5 mutants showed variable levels of susceptibility to LukED that can be classified into 3 groups—no loss of function (Fig. 5A), mild loss of function (Fig. 5B), and severe loss of function (Fig. 5C). Maraviroc (MVC) is a small-molecule entry inhibitor of CCR5-tropic HIV-1 and was found to block LukED-mediated cytotoxicity on CCR5-expressing cells (5, 22). Interestingly, while Y37, Y108, and I198 are critical for MVC binding to CCR5 (23), alanine mutations at these locations have a minimal effect on LukED cytotoxicity (Fig. 5A and B). Mutations located on ECL2 (K171 to E172, and S179) and helix VII (R274 and D276) of CCR5 protected the cells from LukED intoxication, further demonstrating that ECL2 is required for LukED binding and cytotoxicity (Fig. 5C).

### LukED binding does not prevent CCR5-tropic HIV-1 infection.

**FIG 6:**
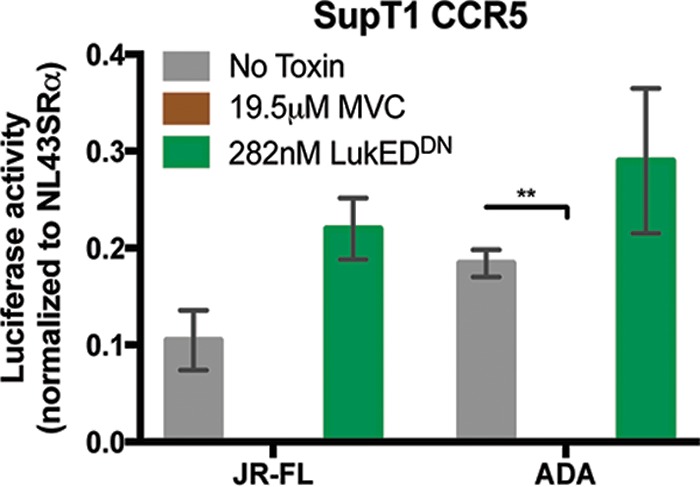
LukED does not inhibit HIV-1 infection of CCR5-expressing cells. SupT1 cells transduced to express CD4 and CCR5 were preincubated with LukE in complex with LukD^DN^ (LukED^DN^), maraviroc (MVC), or no toxin, followed by incubation with CCR5-tropic pseudotyped reporter virus JR-FL and ADA or CXCR4-tropic pseudotyped reporter virus NL43SRα. Bars represent the mean levels of luciferase activity normalized to the NL43SRα control ± SEM (*n* = 3 independent experiments; each experiment was done in triplicate). Statistical analysis was performed using two-way ANOVA with Dunnett’s multiple comparison. **, *P* < 0.01.

To further demonstrate that CCR5-tropic HIV-1 and LukED target CCR5 differently, we designed an experiment where we used the pore formation-deficient mutant LukED^DN^ to compete with gp120-mediated CCR5-tropic HIV-1 fusion. We reasoned that if gp120 and LukED bind to CCR5 differently, then LukED^DN^ would not prevent infection of CCR5-positive SupT1 cells by CCR5-tropic JR-FL- and ADA-pseudotyped HIV-1 luciferase reporter viruses. Our results indicated that, in contrast to MVC, LukED^DN^ did not block the infection (Fig. 6). These data demonstrate that, although CCR5-tropic HIV-1 and LukED share some characteristics in targeting CCR5, they interact with CCR5 differently.

### LukE uses residues 64 to 69 to mediate cytotoxicity on CCR5.

**FIG 7:**
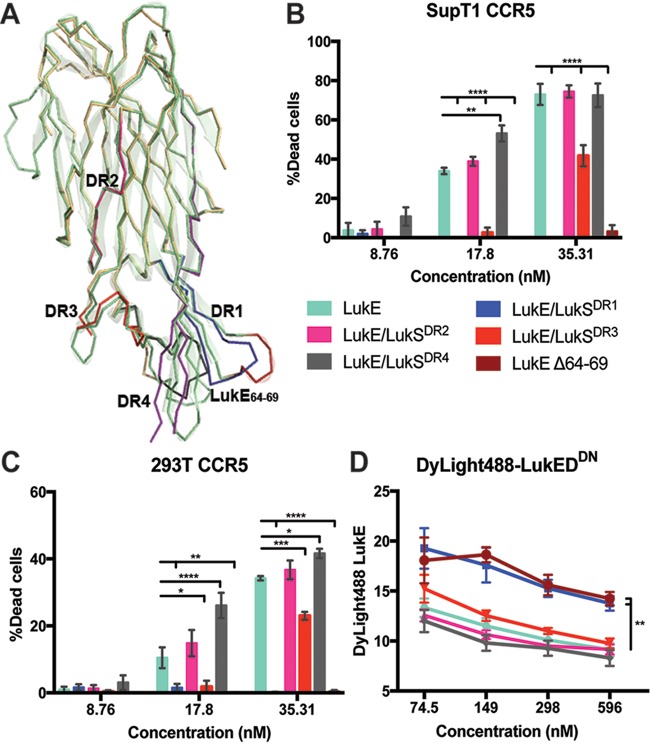
Residues 64 to 69 of LukE are required for LukED cytotoxicity on CCR5-expressing cells. (A) Ribbon diagram of the LukE crystal structure (PDB 3ROH) (51) (in green) overlaid with the LukS-PV crystal structure (PDB 1T5R) (in yellow) (27). Blue, divergent region 1 (DR1) (residues 57 to 75); pink, DR2 (residues 140 to 150); red, DR3 (residues 164 to 178), gray, DR4 (residues 182 to 196); brown, residues 64 to 69. (B) Viability of SupT1 cells expressing WT CCR5 following incubation with increasing concentrations of WT LukED or LukED mutants. Cell viability was determined by CellTiter assay. *n* = 3; data represent mean percentages of dead cells ± SEM. (C) Viability of HEK293T cells expressing WT CCR5 following incubation with increasing concentrations of LukED or LukED mutants. Cell viability was determined by membrane permeability using eFluor450 fixable viability dye. *n* = 3; data represent mean percentages of dead cells ± SEM. (D) Membrane association of DyLight488-LukE (595 nM) in the presence of increasing concentrations of unlabeled WT or mutant LukE and a constant concentration of LukD^DN^ (565 nM). *n* = 3; data represent MFI of DyLight488-LukE ± SEM. Statistical analyses were performed using two-way ANOVA with Dunnett’s multiple comparison. **, *P* < 0.01. LukE/LukS^DR1^, LukE toxin containing DR1 from LukS-PV; LukE/LukS^DR2^, LukE toxin containing DR2 from LukS-PV; LukE/LukS^DR3^, LukE toxin containing DR3 from LukS-PV; LukE/LukS^DR4^, LukE toxin containing DR4 from LukS-PV; LukE Δ64-69, LukE containing in-frame deletion of residues 64 to 69.

CCR5 is one of four receptors targeted by LukED (5, 24, 25). After identifying the regions within CCR5 that support LukED interaction, we next defined the regions in LukE involved in CCR5 targeting. LukE shares 71.1% amino acid identity with LukS-PV; however, while LukE targets CCR5 (5), LukS-PV targets C5aR and C5L2 (26). These subunits recognize the receptors via loops within the cell targeting rim domain (27–29). To identify regions on LukE that recognize CCR5, we employed a loss-of-function approach where the loops within the LukE rim domain were swapped with the corresponding divergent regions (DRs) of the LukS-PV (LukS) rim domain (Fig. 7A; see also Fig. S3) (24). We hypothesized that when the critical LukE rim domains responsible for CCR5 binding are swapped with the corresponding domains of LukS, the binding of the resultant chimeric LukE to CCR5 will be impaired, resulting in reduced LukED-mediated cytotoxicity. Using the chimeric toxins to intoxicate CCR5-expressing cells, we found that LukE/LukS^DR2^ and LukE/LukS^DR4^ showed cytotoxic activity similar to that of the WT toxin (Fig. 7B and C). Intoxication with LukE/LukS^DR3^ partially decreased cytotoxicity (Fig. 7B and C), while the LukE/LukS^DR1^ toxin was fully impaired in its ability to target CCR5-expressing cells for lysis (Fig. 7B and C).

DR1 is the most variable loop between the rim domains of LukE and LukS-PV (Fig. S3A) (24). In particular, LukE contains an extended amino acid sequence, KGSGYE (residues 64 to 69 of the mature, secreted toxin) (Fig. S3A). To investigate the role of these amino acids in targeting CCR5, we generated an in-frame deletion mutant lacking these residues (LukE Δ64–69). The mutated LukE was produced and purified (Fig. S3B), and its activity was evaluated as described above. These experiments revealed that deletion of amino acids KGSGYE phenocopied the lack of activity exhibited by the chimeric toxin LukE/LukS^DR1^ (Fig. 7B and C).

To further evaluate the role of the LukE DR1 and the KGSGYE domain in targeting CCR5, we performed a competition assay to measure the binding of DyLight488-LukE to CCR5-positive cells in the presence of unlabeled LukE or LukE mutants. Strong competition was observed in WT LukE, LukE/LukS^DR2^, LukE/LukS^DR3^, and LukE/LukS^DR4^, whereas LukE/LukS^DR1^ and LukE Δ64–69 showed impairment in this assay (Fig. 7D). These findings demonstrated that residues 64 to 69 in DR1 are critical for LukE targeting of CCR5.

## DISCUSSION

In this study, we used CCR5/CCR2b chimeric receptors to elucidate the molecular basis of the interaction between LukED and CCR5. We identified ECL2 and ECL3 of CCR5 as necessary and sufficient for LukED-mediated cytotoxicity, while analysis of the chimeric receptors showed that ECL2 of CCR5 is necessary but not sufficient to mediate CCR5-tropic HIV-1 fusion by gp120. Using CCR5 point mutants, we identified amino acids on ECL2 and helix VII that are required for LukED cytotoxicity on CCR5-expressing cells. Moreover, using LukE/LukS-PV chimeric toxins, we determined that LukE uses residues 64 to 69 in DR1 for binding to CCR5. Taken together, our findings highlight the fact that LukED and gp120 both target CCR5 but have evolved to use different determinants on the receptor.

CCR5 is expressed by natural killer cells, CD4^+^ T cells, CD8^+^ T cells, regulatory T cells, macrophages, and dendritic cells (30), all of which contribute to the host defense against microbes. Therefore, it is not surprising that CCR5 is targeted by several different microbes, including *Leishmania major* (31) and poxvirus (32), to gain entry to the host. CCR5 was shown to be beneficial for the host immune responses to West Nile virus (33, 34), tickborne encephalitis virus (35), and *Toxoplasma gondii* (36), highlighting its multifaceted role in host defense and pathogenesis. The complex role of CCR5 in host-pathogen interactions is still not fully understood.

MVC, the small-molecule entry inhibitor of CCR5-tropic HIV-1, was demonstrated to block both HIV infection (22) and LukED intoxication (5). MVC blockade of CCR5-tropic HIV-1 entry occurs through noncompetitive allosteric binding of MVC to CCR5 (37, 38). Our study showed that while LukED and HIV shared some characteristics in binding to CCR5 (e.g., the common usage of ECL2), LukED does not prevent HIV infectivity, thus demonstrating that they interact with CCR5 differently. Based on these observations, LukED seems to bind to CCR5 by recognizing structural determinants on ECL2 and ECL3. We speculate that MVC-treated CCR5-expressing cells are protected from LukED intoxication because, upon binding to CCR5, MVC changes the receptor to a conformation that is unrecognizable by LukED via a mechanism that is similar to the allosteric inhibition of gp120 entry observed in CCR5-tropic HIV-1. However, the mode of action by which MVC acts on LukED remains to be tested.

Six leukocidins have been identified in *S. aureus*: LukED, LukSF-PV (or Panton-Valentine leukocidin [PVL]), HlgAB, HlgCB, LukAB, and LukMF′ (39). They target and kill host immune cells and thus are thought to play crucial roles in promoting bacterial survival *in vivo*. Five of the leukocidins target G protein-coupled receptors (5, 24–26, 40, 41), while LukAB targets the integrin component CD11b, to kill host immune cells (42). However, *S. aureus* leukocidin and host receptor interactions are only beginning to be characterized. Previous studies have revealed that LukS-PV and HlgC target C5aR and C5L2 through the recognition of specific extracellular domains on the receptors (43), LukAB targets the I-domain of CD11b (42), and LukMF′ targets ECL2 and ECL3 of bovine CCR1 (41). Together, these studies explain some of the toxin-receptor specificity observed *in vivo*.

The leukocidins share high protein sequence identity, and yet they are specific for disparate receptors and kill different immune cell types (44). For LukED, DR4 was shown to be required for LukE binding and recognition of CXCR1 and CXCR2 (24), and in this study, we demonstrated that DR1 is critical for the binding and recognition of CCR5. Thus, specific LukE DR loops are responsible for receptor recognition. Consistent with this notion, tyrosine-184 in DR4 and tyrosine-250 in DR5 of LukS are important for the binding and cytotoxic activity of LukSF-PV with respect to the C5a receptor (45). In another leukocidin, LukA, glutamic acid-323 is required for receptor binding and toxin activity (46). The interactions between the leukocidins and their receptors are analogous to a lock and a key—the leukocidins require specific domains for the binding to and recognition of specific receptors, while specific regions on the receptors are essential for optimal toxin binding.

The leukocidins are critical virulence factors for *S. aureus* pathogenesis (24, 47–50), and we are only now beginning to understand the molecular basis on which they interact with their receptors. Elucidating how these leukocidins interact with host receptors could provide a foundation for the development of novel inhibitors to combat this important human pathogen.

## MATERIALS AND METHODS

### Generation of the chemokine receptors for transfection and transduction.

CCR5/CCR2b chimeric receptors were generated by overlapping PCR using CCR5 and CCR2b cDNAs followed by cloning into the pcDNA3.1(+) vector. Primers used for chimeric receptor generation are listed in Table S1 in the supplemental material. All constructs were confirmed by Sanger sequencing (Genewiz).

### Cell culture.

Human embryonic kidney 293T cells (HEK293T; ATCC CRL-3216) and SupT1 cells (ATCC CRL-1942) were maintained at 37°C with 5% CO_2_ in RPMI 1640 medium (Cellgro) supplemented with 10% heat-inactivated fetal bovine serum (FBS), penicillin (Pen; 100 U/ml), and streptomycin (Strep; 0.1 mg/ml). Transduced SupT1 cells expressing CCR5 and CCR5 mutants were maintained in the same media supplemented with 1 μg/ml puromycin. All experiments were conducted within approximately 1 month of thawing of frozen cell stocks.

### Virus preparation.

To generate pLenti viruses containing WT CCR5, CCR5 mutants, or CCR5/CCR2b chimeric receptors, HEK293T cells were cultured in Dulbecco’s modified Eagle medium (DMEM) supplemented with 10% FBS and 1× Pen/Strep. HEK293T cells were cotransfected with pRSV-Rev, pMDL-gagpol, pcVSVg, pLenti construct, and pcDNA6. pcVSV-G expresses a cytomegalovirus (CMV)-driven vesicular stomatitis virus (VSV) envelope glycoprotein (G), and pRSV-Rev expresses a Rous sarcoma virus (RSV) long terminal repeat (LTR)-driven HIV-1 Rev. Viruses were harvested 48 h posttransfection, filtered through 0.45-µm-pore-size filters, and frozen at −80°C in DMEM supplemented with 10% FBS and 1× Pen/Strep.

HIV-1 luciferase reporter viruses pseudotyped with a CCR5-tropic HIV envelope were generated by cotransfecting HEK293T cells with pNL43 luc3 E-R^−^ and HIV envelope (pSV-JRFL or pSV-ADA). As a control, HIV-1 luciferase reporter virus pseudotyped with a CXCR4-tropic HIV envelope was generated by cotransfecting HEK293T cells with pNL43 luc3 E-R^−^ and HIV Srα envelope. Viruses were harvested 48 h posttransfection, filtered through 0.45-µm-pore-size filters, and pelleted through 20% sucrose at 30,000 rpm for 90 min at 4°C in an ultracentrifuge. Virus pellets were resuspended in RPMI medium supplemented with 10% FBS and 1× Pen/Strep and frozen at −80°C. Luciferase reporter viruses were normalized by infection of 5 × 10^4^ SupT1 CCR5 cells using various amounts of virus.

### Transfection of 293T.

HEK293T cells were seeded at 25,500 cells/cm^2^ in a 10-cm^2^ dish overnight, followed by transfection with expression plasmids for the CCR5/CCR2b chimeras the next day. For transfection, 30 μg of plasmid DNA was added to 30 μl of Lipofectamine 2000 (Invitrogen) and incubated for 30 min at room temperature (RT). The complexes were added to HEK293T cells, incubated at 37°C with 5% CO_2_ for 6 to 7 h, and then replaced with fresh media. All experiments were conducted the following day.

### Transduction of SupT1.

SupT1 cells were seeded at 200,000/well in a 6-well plate and spinnoculated with 2 ml of pLenti lentiviral vector stock for vectors expressing WT or mutated CCR5 in the presence of Polybrene (Millipore) (10 mg/ml) at 2,500 rpm for 2 h at 30°C. The virus was then removed, and fresh medium was added. Cells were selected 3 days later in RPMI media containing puromycin (1 µg/ml). To detect transduced CCR5, cells were stained with mouse anti-hemagglutinin (anti-HA) antibody (clone 16B12; Covance) (1:1,000) in fluorescence-activated cell sorting (FACS) buffer (1× phosphate-buffered saline [PBS]–2% heat-inactivated FBS) for 1 h at RT. Then, cells were washed twice with FACS buffer and incubated with phycoerythrin-conjugated goat anti-mouse IgG (BioLegend) (1:100) in FACS buffer for 1 h at RT. Cells were washed twice and resuspended in FACS buffer to analyze expression of transduced CCR5 using flow cytometry (LSRII with FACSDiva software; BD).

### Chimera and SupT1 HIV infection.

SupT1 stable cell lines were seeded at 50,000/well in a 96-well plate and spinnoculated with 1.8 ng of p24 from CCR5-tropic luciferase reporter viruses in the presence of Polybrene (8 µg/ml) at 2,500 rpm and 30°C for 2 h. The medium was then changed, and infectivity was determined 3 days later by luciferase assay and read on an Envision 2103 multilabel reader (PerkinElmer).

### Toxin purification from *S. aureus*.

WT LukE and chimeric LukE, LukD, and LukD^DN^ toxins were generated as indicated previously (19, 24). To generate *lukE* Δ64–69, primers VJT629 and VJT684 and primers VJT683 and VJT1114 were used to generate an in-frame deletion of amino acids 64 to 69 of *lukE* by overlapping extension PCR. The final PCR product was amplified using primers VJT629 and VJT1114 and then cloned into the pOS1-*P*_*lukAB*_-*lukA*^ss^-6×His plasmid using BamHI and PstI restriction sites as described previously (19). The purified plasmid was transformed into *Escherichia coli* DH5α competent cells, selected by ampicillin resistance (100 µg/ml), and confirmed by colony PCR and sequencing (Genewiz). The plasmid from a positive clone was purified and electroporated into *S. aureus* RN4220 and selected for by chloramphenicol (10 µg/ml) resistance, and then the plasmid from RN4220 was purified and electroporated into *S. aureus* Newman Δ*lukED hlgACB*::*tet lukAB*::*spec hla*::*ermC* (ΔΔΔΔ) and selected for by chloramphenicol (10 µg/ml) resistance.

Toxins were purified as described previously (19, 24). Briefly, *S. aureus* Newman ΔΔΔΔ strains harboring plasmids with the respective leukocidin sequences were grown overnight in 5 ml tryptic soy broth (TSB; Fisher) supplemented with chloramphenicol (10 μg/ml) at 37°C with shaking at 180 rpm and then subcultured the following day at a 1:100 dilution in TSB supplemented with chloramphenicol (10 μg/ml) and incubated for 5 h at 37°C with shaking at 180 rpm. The cultures were centrifuged for 15 min at 6,000 rpm and 4°C and the supernatants filter sterilized through a 0.22-μm-pore-size filter (Corning). The filtrates were incubated in the presence of a final concentration of 10 mM imidazole and nickel-nitrilotriacetic acid (Ni-NTA) agarose resin (Qiagen) equilibrated with 10 mM imidazole (Fisher) in 1× Tris-buffered saline (TBS; Cellgro) for 30 min at 4°C while nutating. The filtrates were passed through a glass column by gravity filtration, and then Ni-NTA-bound toxins were washed with 25 mM imidazole, followed by a secondary wash with 1× TBS. The Ni-NTA-bound toxins were eluted using 500 mM imidazole. The eluted toxins were dialyzed into 10% glycerol–1× TBS for storage at −80°C. When required, the toxins were concentrated using concentrator columns (Ultra-15 centrifugal filter units; EMD Millipore Amicon) (10,000 nominal molecular weight limit [NMWL], 15-ml capacity) before measurement of the protein concentration was performed using absorbance at 280 nm with a NanoDrop spectrophotometer (Thermo Scientific) and the Beer-Lambert’s equation. Two micrograms of the purified proteins was separated by SDS-PAGE at 90 V for 120 min, followed by Coomassie blue staining to visualize proteins to confirm purity.

### Cytotoxicity assays.

To evaluate the viability of HEK293T and SupT1 cells after intoxication by the leukocidins *in vitro*, cells were seeded at 1 × 10^5^ cells/well in RPMI 1640 without phenol red (Gibco) and supplemented with 10% heat-inactivated fetal bovine serum (FBS; Gemini Bio-Products) in the presence of the indicated concentrations of leukocidins for 2 h at 37°C and 5% CO_2_.

To measure cell viability by FACS analysis, the cells were washed in FACS buffer (1× PBS–2% heat-inactivated FBS–0.05% sodium azide) after intoxication and then incubated with mouse anti-HA antibody (clone 16B12; Covance) (1:1,000) for 30 min on ice. The cells were washed twice on ice in FACS buffer and centrifuged for 5 min at 1,500 rpm and 4°C, followed by incubation of phycoerythrin goat anti-mouse IgG (BioLegend) (1:200) for 30 min on ice. The cells were washed twice on ice in 1× PBS and centrifuged for 5 min at 1,500 rpm and 4°C, followed by incubation with eFluor450 fixable viability dye (eBiosciences) (1:1,000) for 30 min on ice. The cells were fixed in 50 μl FACS fixation buffer (FACS buffer–2% paraformaldehyde). Cell viability was analyzed using flow cytometry (LSRII with FACSDiva software; BD); data are shown as percentages of the total receptor-positive cells that were permeable.

Cell viability was also measured using the CellTiter 96 AQueous ONE solution cell proliferation assay (Promega) when indicated. CellTiter was added to cells at a final concentration of 10% per well and incubated for 2 h at 37°C and 5% CO_2_. Absorbance at 492 nm was measured using an EnVision 2103 multilabel reader (PerkinElmer).

### Binding assays.

For binding assays, HEK293T cells expressing WT CCR5 or chimeric receptors were seeded at 1 × 10^5^ cells/well followed by incubation of mouse anti-HA antibody (clone 16B12; Covance) (1:1,000) for 30 min on ice. The cells were washed twice on ice in FACS buffer and centrifuged for 5 min at 1,500 rpm and 4°C, followed by incubation with allophycocyanin anti-mouse IgG (BioLegend) (1:200) for 30 min on ice. The cells were washed twice on ice in FACS buffer and centrifuged for 5 min at 1,500 rpm and 4°C. The cells were then resuspended in increasing concentrations of DyLight488-LukE in complex with either WT LukD or LukD^DN^ and incubated for 30 min on ice. The cells were centrifuged for 5 min at 1,500 rpm and 4°C, washed on ice in FACS buffer, and centrifuged again for 5 min at 1,500 rpm and 4°C, followed by fixing in 50 μl FACS fixation buffer. Fluorescence of cell-bound toxins was analyzed using flow cytometry (LSRII with FACSDiva software; BD); data are shown as median fluorescence intensity (MFI) of receptor-positive cells.

### Competition assays—LukE interaction with target cells.

For competition assays, HEK293T cells expressing WT CCR5 were seeded at 1 × 10^5^ cells/well followed by incubation of mouse anti-HA antibody (clone 16B12; Covance) (1:1,000) for 30 min on ice, and the cells were washed twice on ice in FACS buffer and centrifuged for 5 min at 1,500 rpm and 4°C, followed by incubation with allophycocyanin goat anti-mouse IgG (BioLegend) for 30 min on ice. The cells were washed twice on ice in FACS buffer and centrifuged as before. The cells were then resuspended in a constant concentration of DyLight488-labeled WT LukE and the pore-formation defective LukD mutant and increasing concentrations of WT LukE or mutant LukE, and LukD^DN^. The cells were incubated for 30 min on ice. Cells were centrifuged for 5 min at 1,500 rpm and 4°C, washed on ice in FACS buffer, and centrifuged again for 5 min at 1,500 rpm and 4°C, followed by fixing in 50 μl FACS fixation buffer. Fluorescence of cell-bound toxins was analyzed using flow cytometry (LSRII with FACSDiva software; BD), where data are shown as median fluorescent intensity (MFI) of receptor-positive cells.

### Competition assay with LukED and blockade of HIV infectivity.

SupT1-CCR5 cells were seeded at 50,000/well on a round-bottom 96-well plate in RPMI media supplemented with 10% heat-inactivated FBS, penicillin (100 U/ml), streptomycin (0.1 mg/ml), and 1 μg/ml puromycin. Cells were treated with 282 nM LukED^DN^, 19.5 μM maraviroc (MVC), or PBS for 30 min. Then, cells were spin-infected with 100 µl of JRFL-, ADA-, or NL43SRα-pseudotyped luciferase reporter viruses at 2,200 rpm and 30°C for 2 h. The medium was changed, and the infectivity was determined 3 days later by luciferase assay using an Envision 2103 multilabel reader (PerkinElmer).

### Graphical and statistical analyses.

Analyses of flow cytometric data were performed using FlowJo (Tree Star Software). Statistical significance was determined using Prism 7.0 (GraphPad Software, Inc.), with two-way analysis of variance (ANOVA) performed with Dunnett’s multiple comparison.

## SUPPLEMENTAL MATERIAL

Figure S1  Surface staining of HEK293T cells transfected to overexpress (A) CCR5 chimeric receptors and (B) CCR2 chimeric receptors. Surface receptor expressions were detected using an anti-HA monoclonal antibody against the HA tag at the N terminus of the chimeric receptors. The histogram depicts transfections from a representative experiment; *n* = 3. Download Figure S1, TIF file, 2.7 MB

Figure S2  Surface staining of SupT1 cells expressing CCR5 mutants. Surface receptor expression was detected using an anti-HA monoclonal antibody against the HA tag at the N terminus of the CCR5 mutant receptors. The histogram depicts transfections from a representative experiment; *n* = 3. Download Figure S2, TIF file, 1.4 MB

Figure S3  (A) Multiple-sequence alignments of LukE and LukS by DNAStar using Clustal W algorithm. Blue indicates DR1, pink indicates DR2, red indicates DR3, gray indicates DR4, and brown indicates residues 64 to 69. (B) Two micrograms per lane of purified chimeric toxins visualized by Coomassie blue staining on a 12% SDS-PAGE gel. Download Figure S3, TIF file, 21.9 MB

Table S1  List of primers used for constructing CCR5/CCR2b chimeric receptors, CCR5 mutants, and LukE Δ64–69. Table S1, XLSX file, 0.03 MB
