# Supplementary figures and images for: Staphylococcus aureus Leukocidin LukED and HIV-1 gp120 Target Different Sequence Determinants on CCR5
Source: mBio. 2016 Dec 13;7(6):e02024-16. doi: 10.1128/mBio.02024-16 (PMC5156306; doi:10.1128/mBio.02024-16)

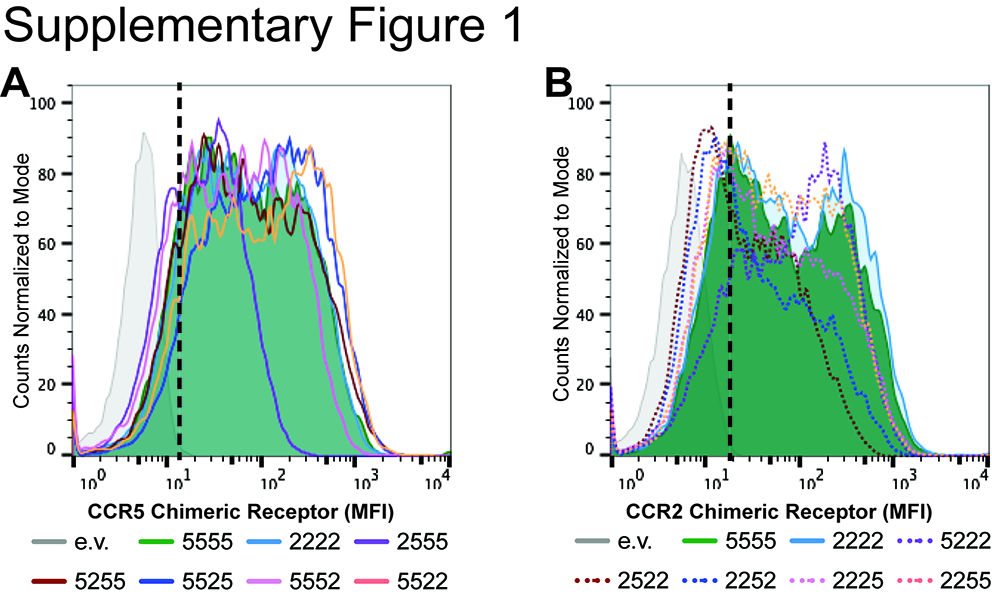

Supplement: Figure S1 — Surface staining of HEK293T cells transfected to overexpress (A) CCR5 chimeric receptors and (B) CCR2 chimeric receptors. Surface receptor expressions were detected using an anti-HA monoclonal antibody against the HA tag at the N terminus of the chimeric receptors. The histogram depicts transfections from a representative experiment; n = 3. Download [file mbo006163098sf1.tif]

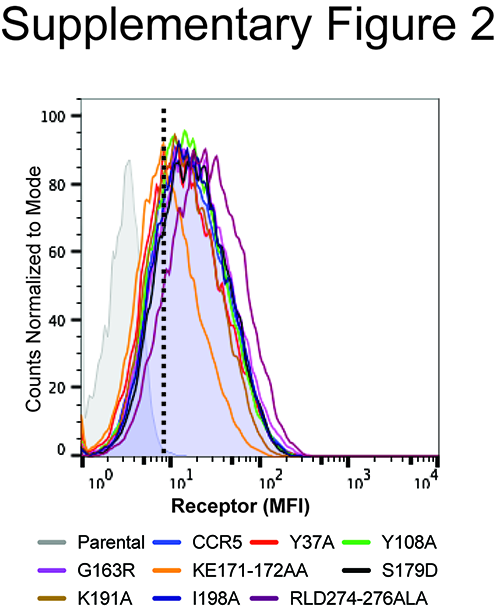

Supplement: Figure S2 — Surface staining of SupT1 cells expressing CCR5 mutants. Surface receptor expression was detected using an anti-HA monoclonal antibody against the HA tag at the N terminus of the CCR5 mutant receptors. The histogram depicts transfections from a representative experiment; n = 3. Download [file mbo006163098sf2.tif]

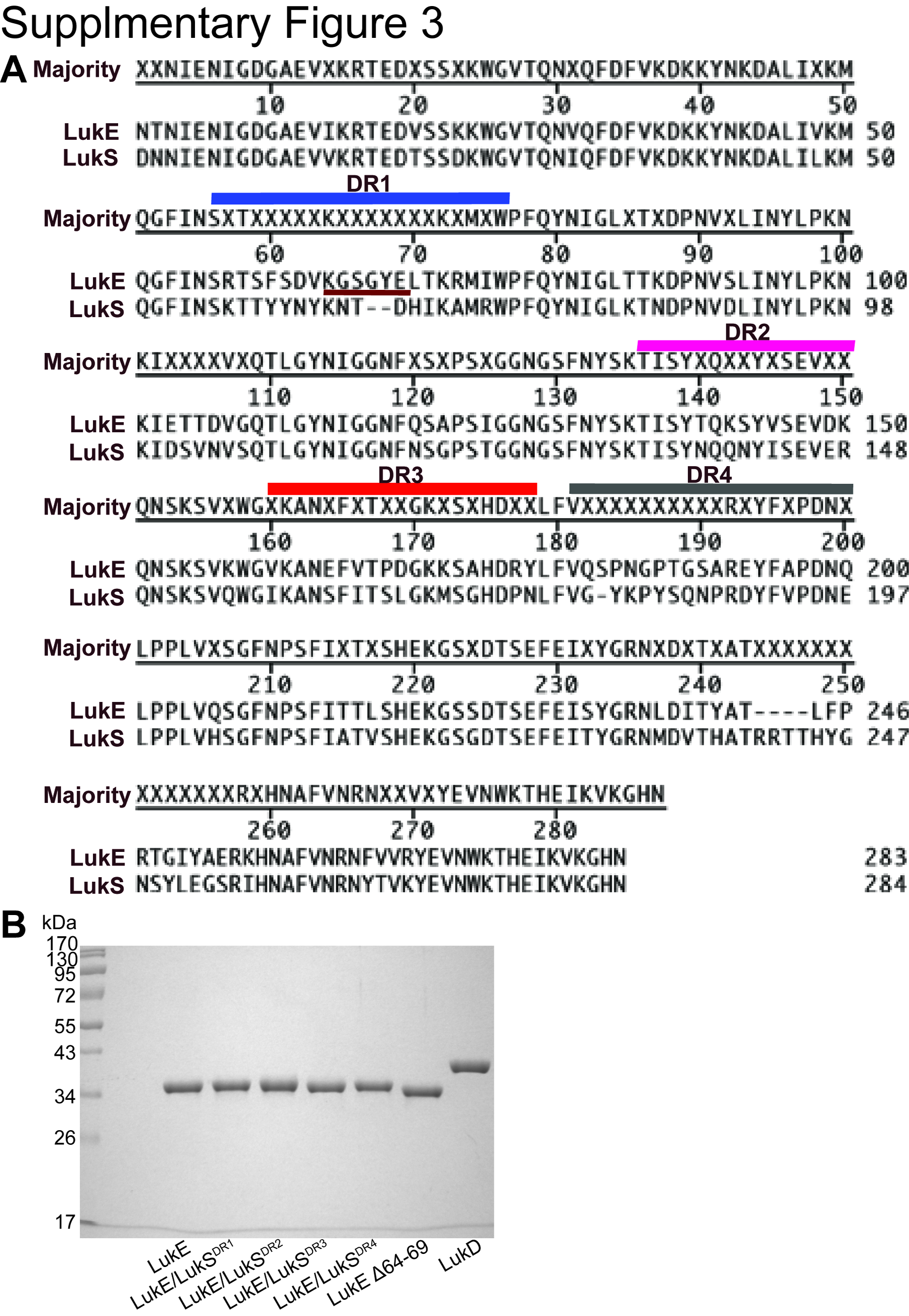

Supplement: Figure S3 — (A) Multiple-sequence alignments of LukE and LukS by DNAStar using Clustal W algorithm. Blue indicates DR1, pink indicates DR2, red indicates DR3, gray indicates DR4, and brown indicates residues 64 to 69. (B) Two micrograms per lane of purified chimeric toxins visualized by Coomassie blue staining on a 12% SDS-PAGE gel. Download [file mbo006163098sf3.tif]
